# Resistive Switching Behavior of Sol–Gel-Processed ZnMgO/ZnO Bilayer in Optoelectronic Devices

**DOI:** 10.3390/nano15171353

**Published:** 2025-09-03

**Authors:** Hee Sung Shin, Dong Hyun Kim, Donggu Lee, Jaehoon Kim

**Affiliations:** 1 Department of Electronic Engineering, Gachon University, Seongnam-si 13120, Gyeonggi-do, Republic of Korea; 2 Department of Semiconductor Engineering, Gyeongsang National University, Jinju-si 52828, Gyeongsangnam-do, Republic of Koreadglee@gnu.ac.kr (D.L.)

**Keywords:** ZnO, ZnMgO, ZnMgO/ZnO, bilayer, resistive switching, sol–gel

## Abstract

Sol–gel-processed zinc oxide (ZnO) and magnesium-doped zinc oxide (ZnMgO) are widely used in quantum dot light-emitting diodes (QLEDs) due to their excellent charge transport properties, ease of fabrication, and tunable film characteristics. In particular, the ZnMgO/ZnO bilayer structure has attracted considerable attention for its dual functionality: defect passivation by ZnMgO and efficient charge transport by ZnO. However, while the effects of resistive switching (RS) in individual ZnO and ZnMgO layers on the aging behavior of QLEDs have been studied, the RS characteristics of sol–gel-processed ZnMgO/ZnO bilayers remain largely unexplored. In this study, we systematically analyzed RS properties of an indium tin oxide (ITO)/ZnMgO/ZnO/aluminum (Al) device, demonstrating superior performance compared to devices with single layers of either ZnMgO or ZnO. We also investigated the shelf-aging characteristics of RS devices with single and bilayer structures, finding that the bilayer structure exhibited the least variation over time, thereby confirming its enhanced uniformity and reliability. Furthermore, based on basic current–voltage measurements, we estimated accuracy variations in MNIST pattern recognition using a two-layer perceptron model. These results not only identify a promising RS device architecture based on the sol–gel process but also offer valuable insights into the aging behavior of QLEDs incorporating ZnMgO/ZnO bilayers, ITO, and Al electrodes.

## 1. Introduction

Zinc oxide (ZnO) is a multifunctional material that plays a key role in optoelectronic devices, particularly in quantum dot light-emitting diodes (QLEDs) [1,2,3] and has also been applied to fast scintillators and nonlinear optical systems [4,5]. This introduction examines the fundamental properties of these materials, their specific functions in QLED technology, and the enhancements they provide to device efficiency and stability. ZnO is distinguished by its large exciton binding energy of 60 meV [6,7], wide band gap of 3.37 eV and high transparency in the visible wavelength range [8,9]—features that make it highly suitable for a wide range of optoelectronic applications. Its excellent conductivity makes ZnO an ideal candidate for use as an electron transport layer (ETL) in QLEDs, since the high electron mobility of ZnO enables rapid and efficient electron transport, thereby minimizing energy losses and enhancing overall device performance [10,11,12].

Furthermore, magnesium-doped zinc oxide (ZnMgO) enables precise tuning of its energy levels by adjusting the magnesium (Mg) concentration, thereby fine-tuning its properties to better align with the specific requirements of high-performance QLEDs [13,14]. This tunability is critical for optimizing the emission wavelengths of QLEDs, thereby enhancing color control and rendering. In addition, the integration of ZnO and ZnMgO into flexible and transparent display technologies is under active investigation, with the potential to significantly advance the design and functionality of next-generation electronic devices [15,16]. These advancements not only highlight the versatility and potential of ZnO and ZnMgO in next-generation display technologies but also emphasize their contribution to the development of more sustainable and efficient lighting solutions. Ongoing research into these materials continues to drive progress in QLED technology. With advancements in material synthesis and doping techniques, ZnMgO can now be fabricated with unprecedented performance, enabling displays that offer higher efficiency and improved color accuracy [17]. Furthermore, the material stability of these materials is being actively investigated to extend the operational lifetime of QLED devices under diverse conditions [18]. Thus, the ongoing advancements are not only improving QLED performance but are also paving the way for future display and lighting technologies that are brighter, more vibrant, and efficient. As research progresses, the critical roles of ZnO and ZnMgO in enabling sustainable and transformative developments in QLEDs become increasingly evident.

ZnO has also been extensively studied in non-volatile memory (NVM) applications [19,20], where its physical and chemical stability significantly enhances device reliability. In particular, ZnO is extensively researched as a material for resistive switching (RS) devices, where it occupies a critical role due to its electrical properties [21,22,23]. ZnO-based materials are being explored not only as low-cost, environmentally friendly alternatives, but also for their wide resistance window, excellent endurance, long retention time, and rapid programming speeds, supporting the pursuit of sustainable technological development [24]. Owing to these advantages, research and development of ZnO-based RS devices continue to expand, aiming to enable high-performance and energy-efficient electronic systems.

Recent studies have also demonstrated the use of ZnMgO in various RS devices, where it improves the intrinsic electrical properties of ZnO and contributes to more stable RS behavior [25,26]. Specifically, Mg doping is known to effectively modulate the oxygen-related stoichiometry of ZnO, resulting in an improved memory performance [27], contributing to increased data storage density and improved reliability in memory applications. Furthermore, the fabrication process of ZnMgO is as straightforward and scalable as that of ZnO, reinforcing its potential as a promising candidate for next-generation RS devices [28]. Taken together, numerous studies have comprehensively investigated single-layer structures of either ZnO or ZnMgO in QLEDs and RS devices.

More recently, however, increasing attention has been paid to the use of ZnO and ZnMgO in a cascaded bilayer configuration, rather than as single-layer structures, particularly in QLED research [29,30,31]. This is due to persistent challenges associated with using a single layer of ZnO or ZnMgO—particularly when constructing thick ETLs required for top-emitting structures that leverage the microcavity effect [11]. In top-emitting QLEDs, the microcavity effect is critical for achieving high luminance efficiency and emission directionality. This necessitates precise control over device thickness to satisfy optical resonance conditions, often requiring the formation of thick ETLs that still maintain excellent electron transport properties and smooth surface morphology [32]. To address these challenges, ZnMgO/ZnO or ZnO/ZnMgO bilayer ETLs have been employed to harness the complementary advantages of each material [30], as ZnO offers high electron mobility, allowing for greater thickness tunability, while ZnMgO, despite its lower conductivity, provides trap-free surface states that help suppress emission quenching—together forming a stepwise electron injection pathway that facilitates efficient charge transport even at significantly increased ETL thickness. Notably, these bilayer configurations have been shown to outperform single-layer structures in terms of charge balance, surface roughness control, and optical resonance tuning, as demonstrated in multiple QLED studies [29,30,31].

**Figure 1:**
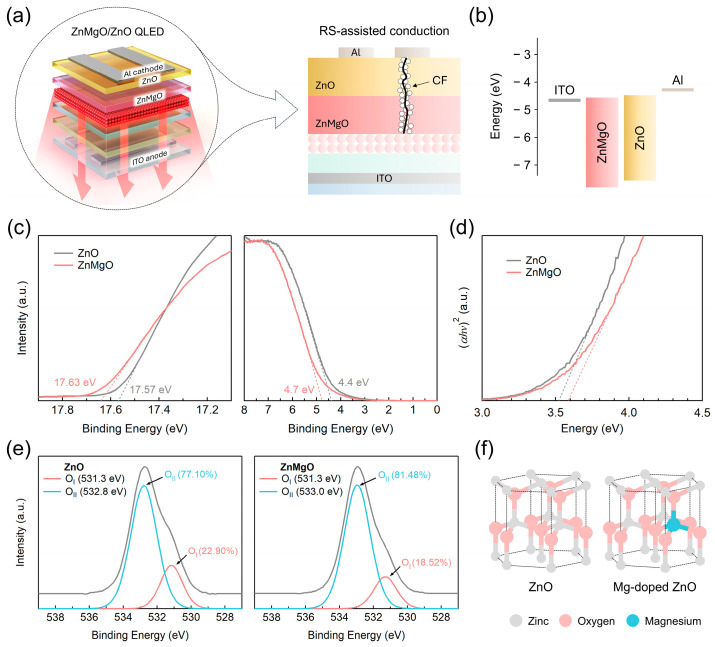
Schematic illustrations of (**a**) ZnMgO/ZnO bilayer-based QLEDs with the RS-assisted conduction mechanism depicted in the right-hand panel; (**b**) energy band diagram; (**c**) UPS spectra showing the secondary cutoff and the Fermi edge region; (**d**) Tauc plot for optical bandgap estimation; (**e**) XPS spectra of ZnO and ZnMgO films with the grey line indicating the overall XPS signal obtained as the sum of sub-peaks; (**f**) lattice structures of ZnO and ZnMgO.

Despite their potential advantages, research on RS devices incorporating ZnMgO/ZnO or ZnO/ZnMgO bilayer structures remains limited. The few existing studies have primarily focused on devices fabricated using physical vapor deposition (PVD) methods, such as pulsed laser deposition [33] and sputtering [34,35], or chemical vapor deposition (CVD) methods, such as aerosol-assisted CVD [36]. While these techniques allow for precise control of film composition and thickness, they generally require vacuum processing and higher costs. The reported RS characteristics from these approaches show relatively stable endurance and switching uniformity; however, they lack scalability for large-area or cost-effective applications. Notably, there is a complete lack of research on bilayer RS devices based on ZnMgO/ZnO fabricated via solution-based techniques, such as sol–gel processing—methods widely adopted in QLED fabrication. Therefore, in this study, we report—for the first time to the best of our knowledge—the fabrication and characterization of sol-gel-processed ZnMgO/ZnO (or ZnO/ZnMgO) bilayer RS devices employing indium tin oxide (ITO) and aluminum (Al) electrodes. While previous studies have focused on achieving stable and efficient RS behavior using methods such as sputtering [34,35], the present work instead emphasizes a solution-processed approach—specifically investigating the RS characteristics of a ZnMgO/ZnO bilayer structure that has been extensively studied in recent QLED architectures [29,30,31]. This investigation is particularly significant not only because the bilayer configuration has demonstrated advantages for RS implementation, but also because recent studies suggest that conduction variations due to conductive filament (CF) formation in ZnO- and ZnMgO-based ETLs during shelf-aging may influence the complex aging behavior of QLEDs (Figure 1a) [37]. Thus, utilizing a sol-gel-processed ZnMgO/ZnO bilayer has the potential to achieve RS device performance comparable to that of conventionally processed devices (Figure 1b), while also offering valuable insights into solution-processed architectures. To support this, we conducted comprehensive analyses, including ultraviolet photoelectron spectroscopy (UPS), X-ray photoelectron spectroscopy (XPS), and ellipsometry on sol-gel-processed ZnO and ZnMgO films. Device-level evaluations were carried out using current–voltage (I–V) measurements, space-charge-limited current (SCLC) [38], and trap-controlled space-charge-limited current (TC-SCLC) analyses [39,40]. Furthermore, we evaluated the recognition accuracy of neural networks (NNs) trained on the Modified National Institute of Standards and Technology (MNIST) dataset [41] using devices based on ZnO, ZnMgO, and ZnMgO/ZnO layers, as a function of shelf-aging. The use of the MNIST dataset in this context serves as a standardized benchmark to assess how well the electrical characteristics of our RS devices, particularly after aging, can support neuromorphic functionalities. To evaluate the neuromorphic potential of our bilayer RS device, we implemented a two-layer perceptron model and conducted MNIST pattern recognition tasks. This system-level demonstration highlights the applicability of ZnO/ZnMgO bilayers—materials traditionally used in optoelectronic devices such as QLEDs—as artificial synapses in NNs. By linking device-level electrical properties with NN-level performance, our study demonstrates the interdisciplinary potential of oxide-based bilayer structures. This approach broadens the applicability of optoelectronic materials to emerging fields such as non-volatile memory and neuromorphic computing. In addition, we present preliminary insights into trilayer RS devices incorporating a ZnMgO/ZnO/ZnMgO configuration, which further underscores the effectiveness and sufficiency of the simpler bilayer structure in achieving optimal device performance. Overall, this research fosters interdisciplinary integration by leveraging knowledge from the QLED domain to advance the development of RS devices. Moreover, it highlights sol-gel-processed ZnO and ZnMgO as promising, low-cost alternatives to conventional high-cost fabrication methods, providing valuable guidance for the design of efficient and scalable RS device architectures.

## 2. Materials and Methods

For material synthesis, 2-methoxyethanol (99.8%), zinc acetate dihydrate (98%), magnesium acetate tetrahydrate (98%), and diethanolamine (99%) were purchased from Sigma-Aldrich. The ZnO precursor solution was prepared by dissolving 0.6 g of zinc acetate dihydrate in 6 mL of 2-methoxyethanol, followed by the dropwise addition of 0.1656 mL of diethanolamine. The ZnMgO precursor solution was synthesized using the same procedure, with the addition of 0.1173 g of magnesium acetate tetrahydrate to the mixture. The Mg:Zn molar ratio in the precursor solution was approximately 20%, based on the weighed amounts of magnesium acetate tetrahydrate (0.1173 g) and zinc acetate dihydrate (0.6 g). This composition was chosen in reference to prior studies showing that Mg doping level of 20% can effectively tune the electronic properties of ZnO without compromising film crystallinity or processability [42]. Both ZnO and ZnMgO solutions were stirred overnight at 1000 rpm.

The architecture of the RS devices follows an ITO/dielectric layer/Al structure, where the dielectric layer consists of either ZnO or ZnMgO single layers, or ZnMgO/ZnO and ZnO/ZnMgO bilayer configurations. The device fabrication process was carried out as follows. ITO-patterned glass substrates were first sequentially sonicated in acetone and isopropanol for at least 30 min each. Subsequently, the substrates were treated with ultraviolet ozone (UVO) for more than 10 min to further clean the surface and enhance surface energy. Following substrate treatment, the respective sol–gel solutions were filtered through a 0.2 µm hydrophobic filter, deposited onto the substrates, and spin-coated at 2000 rpm for 40 s. The coated substrates were then thermally annealed at 200 °C for 1 h in ambient air to induce hydrolysis reactions and form the ZnO or ZnMgO layers. For bilayer fabrication, the bottom layer was first annealed under the same conditions (200 °C for 1 h in ambient air), followed by deposition and annealing of the second layer using the same spin-coating and thermal treatment procedure. Based on a prior report [43] and internal optimization, an annealing condition of 200 °C was found to provide a favorable balance between process compatibility, film quality, and electrical performance. Trials at other temperatures outside this range often resulted in poor film formation and unstable switching behavior, along with a substantial decrease in device yield, which prevented consistent electrical characterization. Finally, an Al top electrode with a thickness exceeding 100 nm was deposited via thermal evaporation under a vacuum pressure of 5 × 10^−6^ Torr. After fabrication, the devices were stored in ambient conditions without encapsulation to maximize interaction between atmospheric oxygen and the internal materials of the RS devices. I–V measurements were performed using a Keithley 2450 source meter. The measurement polarity was configured such that the Al electrode functioned as the anode and the ITO electrode as the cathode—meaning the Al electrode was positive and the ITO electrode negative under positive bias. To maintain structural consistency with QLED architectures—where sol–gel-processed ZnO and ZnMgO are widely used as ETLs—we adopted an asymmetric electrode configuration using ITO as the bottom contact and Al as the top contact. This design choice not only reflects the typical structure of QLEDs but also emphasizes the interdisciplinary intent of our study by bridging optoelectronic and memory device platforms. Additionally, ITO offers excellent transparency and conductivity as a bottom electrode, while Al provides a simple and stable top contact for electrical characterization. This configuration allows us to explore RS behaviors in a setting analogous to established optoelectronic devices. To monitor changes in the I_on_/I_off_ ratio during shelf-aging, the voltage sweep range was adjusted accordingly if the set voltage (V_SET_) and reset voltage (V_RESET_) exhibited temporal shifts.

Synaptic functionalities were modeled using an Al top electrode and an ITO bottom electrode, representing the presynaptic and postsynaptic neurons, respectively. The performance of synaptic devices incorporating ZnO, ZnMgO single layers, and a ZnMgO/ZnO bilayer configuration was evaluated by implementing a multilayer perceptron (MLP) model with an additional hidden layer. The model was trained and evaluated using the MNIST dataset of handwritten digits (0–9), implemented via the NeuroSim + simulator and optimized using stochastic gradient descent (SGD) [44]. The NN architecture consisted of 400 input neurons, 100 hidden neurons, and 10 output neurons, resulting in a total of 400,000 synaptic connections. The MNIST dataset included 60,000 training images and 10,000 test images, each with a resolution of 20 × 20 pixels. These images were converted into 400-element voltage input vectors, which were then processed through matrix multiplication with the synaptic weights to generate ten distinct output current values corresponding to the digit classes. Synaptic weights were dynamically adjusted based on an exponential relationship derived from the long-term potentiation (LTP) and long-term depression (LTD) characteristics, calculated using the experimentally obtained maximum and minimum conductance values. In the simulation, LTP and LTD were modeled with nonlinearities of 2.4 and 4.88, respectively. During training, these weights were iteratively updated at each epoch to improve the network’s digit recognition accuracy, as evaluated on the test dataset.

## 3. Results and Discussion

As shown in Figure 1c, the secondary cutoff energies measured by UPS for ZnO and ZnMgO are 17.57 eV and 17.63 eV, respectively. The corresponding Fermi edge positions are −4.4 eV for ZnO and −4.7 eV for ZnMgO. Based on these values, the valence band maximum (VBM) was calculated to be −8.03 eV for ZnO and −8.27 eV for ZnMgO. The slightly deeper VBM values observed in our UPS measurements (−8.03 eV for ZnO and −8.27 eV for ZnMgO) may stem from a combination of factors that have been suggested in a prior study [45], such as instrumental calibration offsets and the presence of oxygen vacancies, both of which can influence the electronic energy levels. The absence of hydroxyl-related surface species in our XPS analysis suggests that surface dipole contributions are likely negligible. Such variations in VBM values have also been widely reported for ZnO-based materials prepared under different processing conditions. Additionally, from the Tauc plots in Figure 1d, the optical bandgaps (*E*_g_) were determined to be 3.51 eV for ZnO and 3.60 eV for ZnMgO. These results were used to construct the energy band diagram shown in Figure 1b.

Elemental analyses of the ZnO and ZnMgO lattices were performed using XPS, as shown in Figure 1e. For pristine ZnO, the O 1s spectrum revealed two distinct components: OI at 531.3 eV, corresponding to lattice oxygen in Zn–O bonds, and O_II_ at 532.8 eV, associated with oxygen related to vacancy sites [46]. Although the exact position of the oxygen peak may vary slightly across different studies, the relative differences in binding energy are consistent. The entire process—from film fabrication to XPS analysis—was carefully managed through vacuum packaging with silica gel and controlled transportation conditions. Consequently, the third oxygen peak, O_III_ (hydroxyl group), commonly reported in the literature [47], was not detected. While O_III_, typically attributed to surface hydroxyl groups, is often reported in the literature, its presence can be highly sensitive to surface treatment and storage conditions [48]. In this study, high-temperature annealing followed by vacuum storage likely suppressed the formation or retention of hydroxyl species, resulting in the absence of a distinct O_III_ peak. The relative peak areas were 22.90% for OI and 77.10% for O_II_. In the case of ZnMgO, the O 1s spectrum similarly exhibited OI at 531.3 eV and O_II_ at a slightly higher binding energy of 533.0 eV, with relative peak areas of 18.52% and 81.48%, respectively. These results indicate a modest increase in the relative concentration of oxygen vacancies upon Mg doping, consistent with previous reports that Mg incorporation promotes vacancy formation by substituting Zn sites [49], as illustrated in Figure 1f.

**Figure 2:**
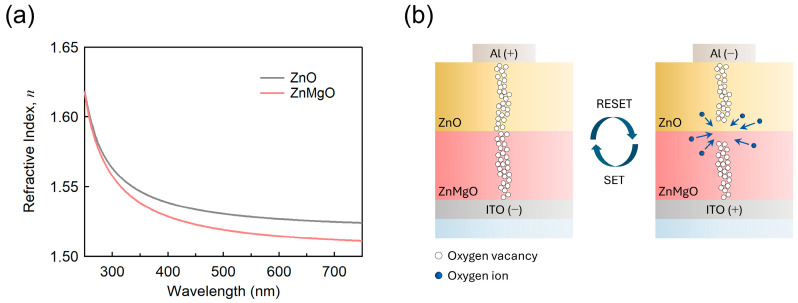
(**a**) Refractive indices of ZnO and ZnMgO films; (**b**) schematic illustration of the SET/RESET processes at the ZnMgO/ZnO interface within the bilayer.

In Figure 2, we explored the origin of the enhanced RS characteristics observed in the ZnMgO/ZnO bilayer structure, focusing on differences in the refractive indices. As previously discussed, Mg doping—where Mg substitutes for Zn—induces the formation of oxygen vacancies [49]. This increase in vacancy concentration led to a reduction in the refractive index of ZnMgO, as shown in Figure 2a. At a wavelength of 550 nm, the refractive index of ZnO is measured to be 1.528, whereas that of ZnMgO is slightly lower at 1.517.

(1) $$E=\frac{Q}{\epsilon_{0}\epsilon_{r}A}$$

(2) $$n^{2}=\epsilon_{r}$$

RS devices can be conceptually modeled as parallel-plate capacitors, where the electric field across the dielectric layer is inversely proportional to the relative permittivity ($\epsilon_{r}$) of the material between the electrodes, as described by Equation (1). Since the refractive index (*n*) and relative permittivity ($\epsilon_{r}$) are related in most non-magnetic materials by Equation (2), it can be inferred that a stronger electric field is established across the ZnMgO layer compared to the ZnO layer when a voltage is applied to the ZnMgO/ZnO bilayer. This is due to the lower relative permittivity ($\epsilon_{r}$) of ZnMgO, as indicated by its lower refractive index. Although the refractive index difference between ZnO (*n* = 1.528) and ZnMgO (*n* = 1.517) at 550 nm appears negligible for optical applications [50], even slight variations in dielectric properties can influence the local electric field distribution in RS devices [51]. When two materials with different permittivities are in contact, such differences may modify the electric field near the interface, thereby affecting the dynamics of filament formation and rupture [52]. This sensitivity is particularly relevant in oxygen-vacancy–based switching mechanisms, where local field strength plays a critical role. Therefore, the observed refractive index contrast, while optically minor, may still be functionally significant in determining the device’s electrical behavior. Consequently, the electric field distribution varies at the ZnO/ZnMgO interface, and the higher concentration of oxygen vacancies in ZnMgO suggests that additional oxygen vacancy-related modifications may also occur at the interface (Figure 1e). Numerous studies on bilayer-based RS devices have reported that the improved switching stability arises from the formation and rupture of CFs predominantly at the interfacial region between the two layers (Figure 2b) [53,54]. This interpretation is consistent with the thin-film characterizations presented in Figure 1e and Figure 2a, as well as with the RS performance of the bilayer devices that will be discussed in the following sections.

**Figure 3:**
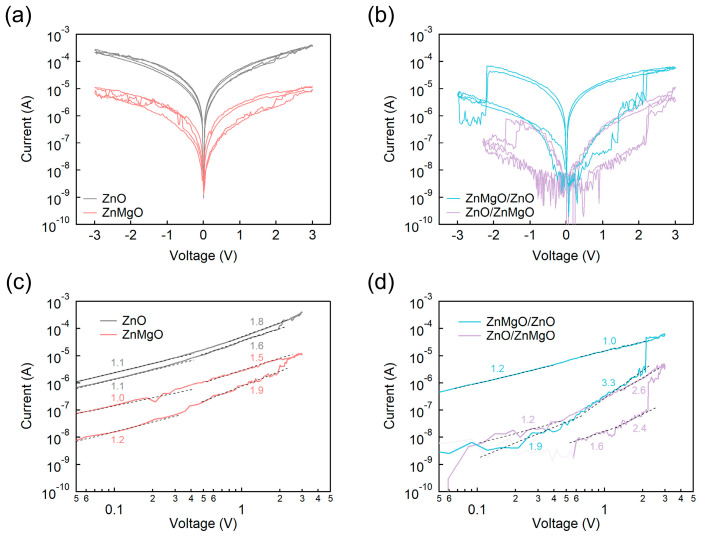
I–V characteristics of RS devices based on: (**a**) ZnO and ZnMgO single layers; (**b**) ZnMgO/ZnO and ZnO/ZnMgO bilayers. Logarithmic I–V plots for: (**c**) ZnO and ZnMgO single layers; (**d**) ZnMgO/ZnO and ZnO/ZnMgO bilayers.

To evaluate the RS properties of the ZnMgO/ZnO bilayer structure, we compared I–V characteristics (Figure 3a,b) and their corresponding logarithmic plots (Figure 3c,d) for devices fabricated with ZnO, ZnMgO, and ZnO/ZnMgO configurations. The ZnO-based RS device exhibited a high resistance state (HRS) current of 5.19 × 10^−6^ A and a low resistance state (LRS) current of 9.88 × 10^−6^ A, resulting in an I_on_/I_off_ ratio of 1.90. *I*_on_ and *I*_off_ represent the current magnitudes in the LRS and HRS, respectively, and were measured at 0.3 V, which is approximately 10% of the maximum voltage range. The ZnMgO-based RS device exhibited an HRS current of 6.63 × 10^−8^ A and an LRS current of 5.74 × 10^−7^ A, resulting in an I_on_/I_off_ ratio of 8.66. The ZnMgO/ZnO bilayer RS device showed an HRS current of 1.42 × 10^−8^ A and a LRS current of 3.33 × 10^−6^ A, yielding an I_on_/I_off_ ratio of 2.35 × 10^2^. In contrast, the ZnO/ZnMgO bilayer device demonstrated an HRS current of 3.20 × 10^−9^ A and an LRS current of 1.96 × 10^−8^ A, corresponding to an I_on_/I_off_ ratio of 6.13. It is evident that bilayer structures—ZnMgO/ZnO and ZnO/ZnMgO—exhibit a broader RS window compared to single-layer devices composed of ZnO or ZnMgO. To assess reproducibility more carefully, we conducted measurements with a wider range of switching cycles, as shown in Figure S1. Among the two bilayer configurations, the ZnMgO/ZnO structure demonstrated a significantly higher I_on_/I_off_ ratio and greater LRS current, despite both configurations displaying comparable HRS current levels. The enhanced current flow in the LRS can be attributed to the cascaded alignment of the conduction band minimums (CBMs) within the ZnMgO/ZnO structure (Figure 1b). In this configuration, the CBMs of ZnMgO and ZnO form an energetically favorable gradient between the ITO and Al electrodes. Specifically, the energy level sequence in the ZnMgO/ZnO device—ITO (−4.7 eV)/ZnMgO (−4.67 eV)/ZnO (−4.52 eV)/Al (−4.2 eV)—promotes more efficient electron transport compared to the reverse configuration in the ZnO/ZnMgO device—ITO (−4.7 eV)/ZnO (−4.52 eV)/ZnMgO (−4.67 eV)/Al (−4.2 eV).

Furthermore, analysis of the logarithmic *I*–*V* characteristics enables identification of the dominant conduction mechanisms in each device. A slope near 1 indicates ohmic conduction, a slope around 2 corresponds to SCLC [38], and slopes exceeding 2 suggest the presence of TC-SCLC [40]. In the case of ohmic conduction, the current is directly proportional to the applied voltage, following Ohm’s law. This linear behavior arises when the active layer exhibits constant resistivity, allowing electrons to flow with minimal scattering or trapping—conditions typically observed in LRS. SCLC arises in RS devices when the injection of charge carriers from the electrodes exceeds the rate of their thermal generation within the semiconductor. Under these conditions, the current deviates from Ohm’s law and becomes limited by the ability of the device to transport the accumulated charge, resulting in a quadratic dependence on the applied voltage. This behavior reflects carrier accumulation and a shortage of free carriers to neutralize the space charge. In TC-SCLC, the current is further influenced by trap states within the material, which capture and release carriers, thereby affecting their mobility and the overall transport dynamics. As a result, the I–V characteristics exhibit multiple slope regions in the logarithmic plot, corresponding to different trap-filling regimes and complex interactions between free and trapped carriers.

As shown in Figure 3c, both the ZnO and ZnMgO single-layer devices initially exhibited ohmic conduction, followed by a transition to SCLC. After switching from HRS to LRS, the conduction mechanism returned to ohmic behavior, again passing through an SCLC regime. In contrast, the bilayer devices in Figure 3d displayed SCLC behavior from the onset of HRS, with slope values of 1.9 for ZnMgO/ZnO and 1.6 for ZnO/ZnMgO—indicating a deviation from ideal ohmic conduction. This behavior is attributed to the increased overall thickness of the bilayer structures, as well as the presence of an interfacial layer, which together promote space-charge effects and suppress ohmic conduction in the HRS. Notably, prior to the transition from HRS to LRS, a region with a slope greater than 2 was observed in the bilayer devices, indicative of TC-SCLC. This behavior suggested the involvement of oxygen vacancy traps in the formation of CFs at the interface. In particular, oxygen vacancies—one of the key intrinsic point defects—are known to significantly affect the electrical behavior of wide bandgap oxides such as ZnO and its alloys. These defects can act as deep or shallow trap states, influencing carrier concentration, charge transport, and recombination dynamics. Extensive studies, including those by Popov et al. and McCluskey et al., have systematically examined the nature, formation, and electronic impact of intrinsic point defects in oxide semiconductors [55,56]. Following this trap-dominated phase, the devices transitioned through SCLC and eventually exhibited ohmic conduction in the LRS. These results demonstrate that bilayer structures more prominently displayed the TC-SCLC phase compared to single layer devices, indicating a more gradual and controlled process of trap formation and rupture—primarily associated with oxygen vacancies. This mechanism is analogous to that observed in optoelectronic devices such as QLEDs, where trap-filling alters the conduction behavior, a phenomenon often described as trap-filled space-charge-limited current (TF-SCLC) [57]. In RS devices, the high electric field facilitates oxygen vacancy generation, leading to CF formation, and drives the transition in conduction mechanism as traps accumulate and are progressively filled during the TC-SCLC phase [58].

**Figure 4:**
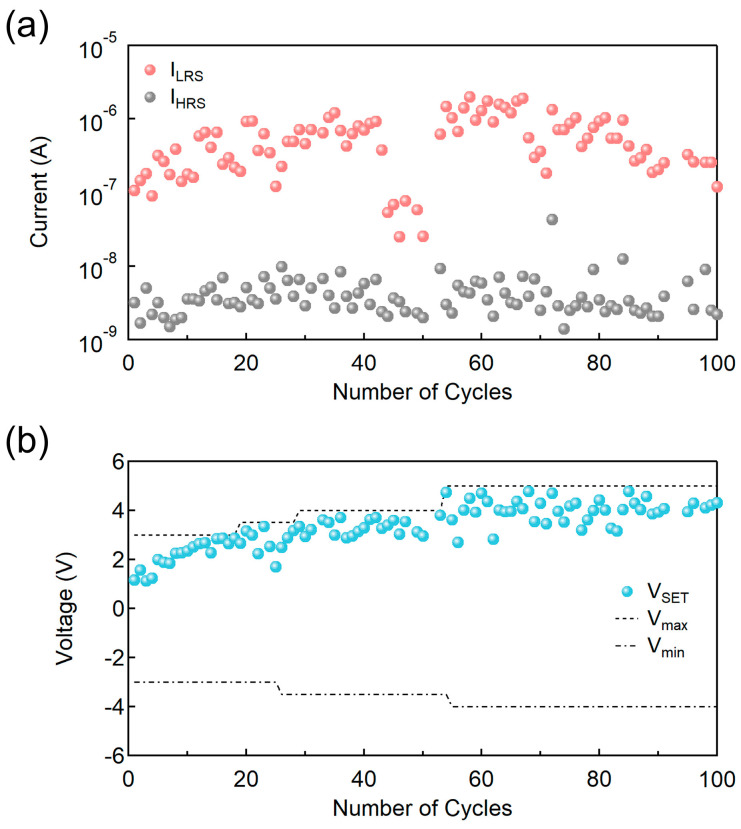
(**a**) Evolution of currents in HRS and LRS at respective V_SET_ values upon number of cycles; (**b**) variation in V_SET_ values alongside changes in maximum (V_max_) and minimum (V_min_) voltage sweep values for continuous RS behavior.

In addition, the cycle-to-cycle reliability of the ITO/ZnMgO/ZnO/Al device—identified as exhibiting the most prominent RS characteristics among the tested configurations—was evaluated, as shown in Figure 4. The device maintained a stable I_on_/I_off_ ratio in the range of approximately 10^2^ to 10^3^ over 100 consecutive switching cycles. While this number of cycles provides initial insight into device endurance, further extended cycling tests (e.g., 1000 cycles) are planned in future work to comprehensively validate long-term stability. Notably, non-uniform trends were observed in both the low resistance state current (I_LRS_) and high resistance state current (I_HRS_) during cycling (Figure 4a). These variations are primarily attributed to the sol–gel process used for oxide layer fabrication, which is susceptible to morphological and compositional fluctuations, leading to device-to-device and cycle-to-cycle variability. The lack of encapsulation is also a relevant factor, especially given the environmental sensitivity of sol–gel–processed structures. Here, I_HRS_ refers to the current in the HRS immediately before the SET transition, and I_LRS_ denotes the current in the LRS at the same voltage level. In addition, a gradual increase in V_SET_ over time was observed (Figure 4b), likely reflecting progressive changes in the switching interface. To account for this drift and ensure consistent observation of RS behavior, the maximum (V_max_) and minimum (V_min_) sweep voltages were progressively expanded during measurement.

**Figure 5:**
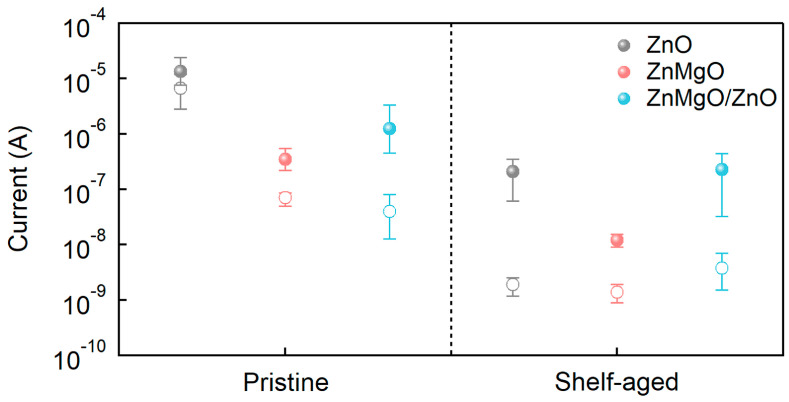
Variation in current values in RS devices with dielectric layers of ZnO, ZnMgO, and ZnMgO/ZnO due to shelf aging at a voltage of 0.3 V. Current measurements are shown for devices in HRS (open circle) and LRS (filled circle). The shelf-aging durations of RS devices were 19 days for ZnO, 22 days for ZnMgO, and 26 days for ZnMgO/ZnO.

Furthermore, Figure 5 presents the current variations at 0.3 V for RS devices incorporating ZnO, ZnMgO, and ZnMgO/ZnO dielectric layers, measured in both HRS (open circle) and LRS (filled circle) after shelf-aging. In this context, shelf-aging refers to the storage of the device under ambient conditions without encapsulation, which is also extensively studied in QLEDs [37,59]. The ZnO, ZnMgO, and ZnMgO/ZnO devices were shelf-aged for 19, 22, and 26 days, respectively, as electrical characterization extended over multiple days and was performed sequentially. Nevertheless, all devices were fabricated under identical conditions on the same day to minimize batch-to-batch variation. As the devices were sufficiently aged prior to measurement, these differences are unlikely to introduce any significant bias in the comparative analysis. The most notable observation was the variation in current levels across both resistance states, which strongly depended on the specific dielectric structure. For the pristine ZnO-based RS device, the average HRS and LRS currents were 6.69 × 10^−6^ A and 1.35 × 10^−5^ A, with observed ranges of 2.79 × 10^−6^–1.19 × 10^−5^ A and 7.60 × 10^−6^–2.42 × 10^−5^ A, respectively. After shelf-aging, the corresponding values decreased to 1.93 × 10^−9^ A and 2.09 × 10^−7^ A, with ranges of 1.18 × 10^−9^–2.53 × 10^−9^ A and 6.09 × 10^−8^–3.46 × 10^−7^ A, respectively. The I_on_/I_off_ ratio increased from 2.02 to 1.08 × 10^2^, indicating a notable enhancement in switching contrast. This observation is consistent with previous studies suggesting that the formation of an interfacial AlO_x_ layer at the ZnO/Al interface can influence charge transport characteristics [59,60]. However, for reliable integration into NN applications, such significant variations in RS characteristics due to aging are generally undesirable [61]. For the ZnMgO-based RS device, the average HRS and LRS currents before aging were 7.14 × 10^−8^ A and 3.50 × 10^−7^ A, with observed ranges of 5.01 × 10^−8^–8.63 × 10^−8^ A and 2.19 × 10^−7^–5.42 × 10^−7^ A, respectively. After shelf-aging, these values declined to 1.39 × 10^−9^ A and 1.22 × 10^−8^ A, with ranges of 8.89 × 10^−10^–1.90 × 10^−9^ A and 9.18 × 10^−9^–1.54 × 10^−8^ A, respectively. Although the I_on_/I_off_ ratio improved from 4.90 to 8.78, both current levels decreased significantly. In contrast, the ZnMgO/ZnO bilayer device exhibited the most stable behavior under shelf-aging, with only modest changes in both HRS/LRS current levels and I_on_/I_off_ ratio. Initially, the average HRS and LRS currents were 4.00 × 10^−8^ A and 1.25 × 10^−6^ A, with observed ranges of 1.27 × 10^−8^–8.07 × 10^−8^ A and 4.54 × 10^−7^–3.32 × 10^−6^ A, respectively. After aging, these values shifted only slightly to 3.79 × 10^−9^ A and 2.27 × 10^−7^ A, with ranges of 1.51 × 10^−9^–7.00 × 10^−9^ A and 3.21 × 10^−8^–4.40 × 10^−7^ A, respectively. The I_on_/I_off_ ratio showed a modest increase from 3.13 × 10^1^ to 5.99 × 10^1^. These results suggest that the ZnMgO/ZnO bilayer structure offers the most stable RS characteristics among the tested configurations, making it a promising candidate for implementation in NN systems, particularly those based on three-dimensional stacked arrays [62].

**Figure 6:**
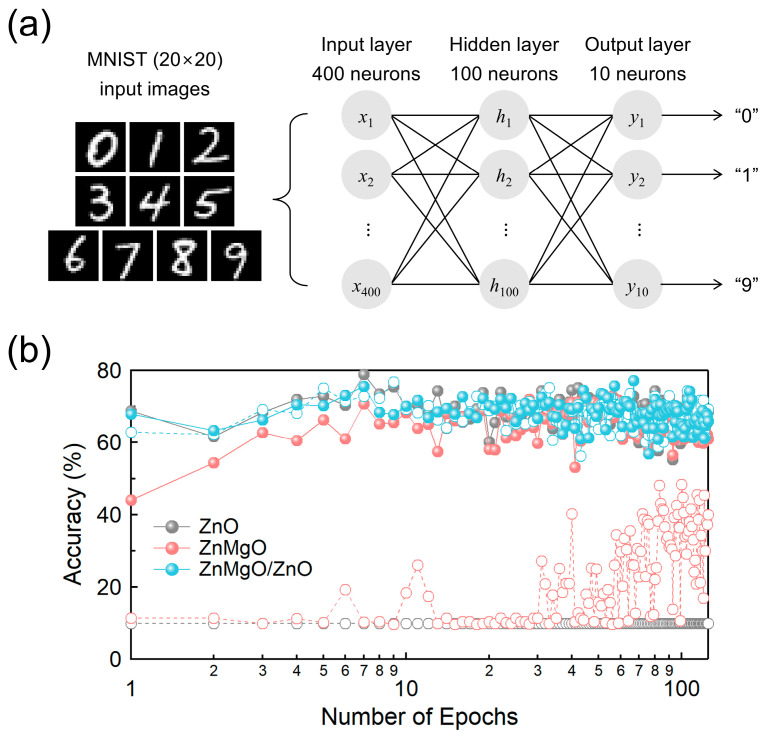
(**a**) Diagram of a two-layer perceptron model and (**b**) accuracy variations as a function of the number of epochs for MNIST pattern recognition using pristine (open circle) and shelf-aged (filled circle) RS devices with ZnO, ZnMgO single layers, and ZnMgO/ZnO bilayer.

The learning capabilities of NNs based on ZnO, ZnMgO, and ZnMgO/ZnO RS devices were simulated as a function of shelf-aging. Multilayer NNs are essential for parallel processing [63], as they facilitate complex interactions between presynaptic and postsynaptic neurons. In Figure 6, we evaluated the variation in NN inference accuracy over 125 training epochs using RS devices based on ZnO, ZnMgO, and ZnMgO/ZnO layers. The respective I_on_/I_off_ ratios from Figure 5 were adopted to simulate the device behavior within the NN. The NN employing pristine ZnO—with a low I_on_/I_off_ ratio of 2.02—maintained a constant accuracy of approximately 10% throughout the training period, indicating a failure to learn beyond random guessing [61]. In contrast, the NN using shelf-aged ZnO, which exhibited a significantly improved I_on_/I_off_ ratio of 1.08 × 10^2^, achieved an initial accuracy of 68.8% and maintained performance within the 60–70% range across epochs, demonstrating a marked improvement in learning capability. Among the tested materials, ZnO exhibited the most significant change in I_on_/I_off_ ratio due to shelf-aging and correspondingly showed the largest variation in NN inference accuracy. For ZnMgO, which had an I_on_/I_off_ ratio of 4.90 prior to shelf-aging, the NN initially demonstrated low accuracy (~10%), similar to the ZnO-based model. However, accuracy gradually improved over training, reaching a range of 30–50% after 125 epochs. This result emphasizes the sensitivity of learning performance to I_on_/I_off_ ratios below 10, with noticeable improvements occurring once this threshold is exceeded—a trend consistent with previous reports [61]. The shelf-aged ZnMgO device, with an I_on_/I_off_ ratio of 8.78, achieved an initial accuracy of 44.1%, which gradually increased during training and eventually reached levels comparable to those of shelf-aged ZnO and both pristine and aged ZnMgO/ZnO bilayer-based NNs, stabilizing in the 60–70% range. In contrast, the NN incorporating the ZnMgO/ZnO bilayer—exhibiting I_on_/I_off_ ratios of 3.13 × 10^1^ before aging and 5.99 × 10^1^ after aging—demonstrated consistently high performance, maintaining an accuracy of 60–70% throughout all training epochs. This stability indicates that the bilayer configuration provides the most robust and reliable learning characteristics, making it a promising candidate for NN implementations.

**Figure 7:**
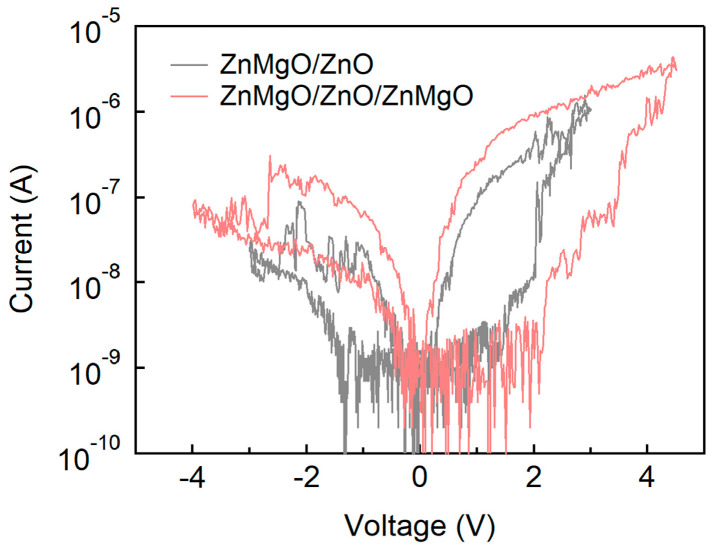
I–V characteristics of RS devices based on ZnMgO/ZnO bilayer and ZnMgO/ZnO/ZnMgO trilayer configurations.

Lastly, the transition from single-layer and bilayer structures to a trilayer configuration was analyzed, as shown in Figure 7. To validate consistency across devices, we included additional *I*–*V* data from repeated measurements, as shown in the overlaid plots in Figure S2. Film thickness measurements using alpha-step profilometry showed average thicknesses of 140 nm (standard deviation 8 nm) for ZnO and 126 nm (standard deviation 10 nm) for ZnMgO, confirming uniformity across the substrates (Figure S3). Based on these results, the thicknesses of the bilayer and trilayer films are presumed to be approximately two and three times that of the single-layer films, assuming consistent deposition parameters. As previously observed in Figure 3, despite the bilayer being nearly twice as thick as the single layer devices, both V_SET_ and V_RESET_ remained within the range of −3 V to +3 V. This observation is consistent with previous studies on bilayered QLEDs, which reported similar operating voltage ranges, suggesting that increased thickness in the bilayer does not significantly impact the driving voltage [30]. Although an increase in dielectric layer thickness would typically contribute to higher V_SET_ and V_RESET_ values, the cascading alignment of CBMs in ZnMgO and ZnO, along with the gradual transition in electrode work functions, enables the bilayer device to operate within the same voltage range as single-layer devices. This band alignment effectively reduces the interfacial energy barriers, facilitating electron transport. However, the trilayer structure requires a broader voltage range of approximately −4 V to +4.5 V for switching, which exceeds that of both the single-layer and bilayer devices. This is attributed to the non-cascading, zig-zag energy level alignment in the ITO/ZnMgO/ZnO/ZnMgO/Al configuration. In this arrangement, the deeper CBM of ZnMgO relative to ZnO disrupts the smooth energy gradient, thereby necessitating higher voltages to support electron transport from the Al electrode to ITO. The increase in total thickness, the presence of non-cascading energy levels, and the addition of multiple interfaces in the trilayer structure are all inferred to contribute to the elevated V_SET_ and V_RESET_ values. While introducing a trilayer configuration may increase the number of interfaces and highlight regions with varying electrical permittivity (*ϵ*), potentially enhancing certain RS characteristics, it also introduces energy level discontinuities and interfacial complexities that require higher operating voltages. Therefore, the ZnO/ZnMgO bilayer structure offers a more straightforward and efficient RS behavior compared to the trilayer, combining favorable band alignment with moderate thickness and fewer interfaces. Among the multilayer configurations evaluated, the bilayer structure demonstrates the most practical and effective RS performance for potential integration into low-power and neuromorphic computing applications.

## 4. Conclusions

In this study, we investigated RS characteristics of devices based on sol–gel-processed ZnO and ZnMgO, with particular emphasis on the ZnMgO/ZnO bilayer structure. For the first time, we demonstrated that RS performance is significantly enhanced in the sol–gel-processed ZnMgO/ZnO bilayer when integrated with Al and ITO electrodes—materials commonly used in QLED devices. Mg doping in ZnMgO led to an increased concentration of oxygen vacancies through substitutional incorporation, resulting in a higher density of vacancies compared to ZnO. Furthermore, ZnMgO exhibited a lower refractive index (*n*) and relative permittivity (ϵ) than ZnO, contributing to the formation of electric field and defect boundaries at the ZnO/ZnMgO interface. These interfacial regions serve as active sites for the formation and rupture of CFs, which are responsible for RS behavior. The ZnMgO/ZnO interfacial structure exhibited more pronounced RS characteristics under pristine conditions compared to single-layer ZnO or ZnMgO devices, primarily due to the stronger influence of TC-SCLC. We further confirmed the stability of I_LRS_ and I_HRS_ across multiple switching cycles and identified a limitation in the form of a gradually increasing V_SET_ over time. Nevertheless, shelf-aging tests revealed that the ZnMgO/ZnO bilayer demonstrated the highest stability and the least variation in current and switching voltage among the structures evaluated, outperforming conventional single-layer RS devices. Finally, using these experimentally derived RS characteristics, we implemented a two-layer perceptron NN model to investigate pattern recognition trends on the MNIST dataset. The ZnMgO/ZnO bilayer structure, which exhibited the least variation in I_on_/I_off_ ratio upon shelf-aging, also demonstrated the most stable and consistent recognition accuracy in NN simulations. In conclusion, this work presents the first report of a sol–gel-processed ZnMgO/ZnO-based RS device and provides valuable insights into the electrical behavior of this bilayer configuration, as well as its conduction mechanisms relevant to QLED device architectures. These findings introduce a new pathway for the development of reliable RS devices and offer meaningful guidance for researchers exploring the shelf-aging behavior of bilayer-based QLEDs.

## Data Availability

The original contributions presented in this study are included in the article; further inquiries can be directed to the corresponding authors.

## Supplementary Materials

The following are available online at https://www.mdpi.com/article/10.3390/nano15171353/s1, Figure S1: Device-to-device reproducibility of resistive switching characteristics: overlaid I–V curves measured from multiple devices based on (a) ZnO (b) ZnMgO (c) ZnMgO/ZnO and (d) ZnO/ZnMgO structures, Figure S2: Device-to-device reproducibility of resistive switching characteristics: overlaid I–V curves measured from multiple devices based on (a) ZnMgO/ZnO bilayer and (b) ZnMgO/ZnO/ZnMgO trilayer structures, Figure S3: Thickness distribution of ZnO and ZnMgO films measured by alpha-step profilometry at multiple evenly distributed points across the substrate.

## References

[1] Kim J. (2024). Recent progresses and challenges in colloidal quantum dot light-emitting diodes: A focus on electron transport layers with metal oxide nanoparticles and organic semiconductors. Nanoscale Horiz..

[2] Ali A., Jiang W., Choi Y., Jeon E., Chae H. (2022). Enhanced charge balance with antibiotics in both electron and hole transport layers of InP/ZnSexS1–x/ZnS-based quantum dot light-emitting diodes. J. Alloys Compd..

[3] Chen T., Yu K., Hu H., Li Y., Huang W., Li R., Qie Y., Lin H., Guo T., Li F. (2025). Engineering Electron Transport Layer with Ionic Liquid for High-Performance Quantum Dot Light-Emitting Diodes. ACS Appl. Nano Mater..

[4] He S., Zhang S., Wang F., Chen L., Li Y., Ruan J., Ouyang X., Du X. (2024). Enhancement of the luminescence intensity of a ZnO:Ga crystal scintillator via coating CsPbBr3 quantum dot films. Opt. Mater..

[5] Uklein A.V., Multian V.V., Kuz’micheva G.M., Linnik R.P., Lisnyak V.V., Popov A.I., Gayvoronsky V.Y. (2018). Nonlinear optical response of bulk ZnO crystals with different content of intrinsic defects. Opt. Mater..

[6] Sun H.D., Makino T., Segawa Y., Kawasaki M., Ohtomo A., Tamura K., Koinuma H. (2002). Enhancement of exciton binding energies in ZnO/ZnMgO multiquantum wells. J. Appl. Phys..

[7] Su Y.Q., Zhu Y., Yong D., Chen M., Su L., Chen A., Wu Y., Pan B., Tang Z. (2016). Enhanced Exciton Binding Energy of ZnO by Long-Distance Perturbation of Doped Be Atoms. J. Phys. Chem. Lett..

[8] Saoud F.S., Plenet J.C., Henini M. (2015). Band gap and partial density of states for ZnO: Under high pressure. J. Alloys Compd..

[9] Wang T., Liu Y., Fang Q., Wu M., Sun X., Lu F. (2011). Low temperature synthesis wide optical band gap Al and (Al, Na) co-doped ZnO thin films. Appl. Surf. Sci..

[10] Yu Y., Liang Y., Yong J., Li T., Hossain M.S., Liu Y., Hu Y., Ganesan K., Skafidas E. (2022). Low-Temperature Solution-Processed Transparent QLED Using Inorganic Metal Oxide Carrier Transport Layers. Adv. Funct. Mater..

[11] Lee T., Hahm D., Kim K., Bae W.K., Lee C., Kwak J. (2019). Highly Efficient and Bright Inverted Top-Emitting InP Quantum Dot Light-Emitting Diodes Introducing a Hole-Suppressing Interlayer. Small.

[12] Kirkwood N., Singh B., Mulvaney P. (2016). Enhancing Quantum Dot LED Efficiency by Tuning Electron Mobility in the ZnO Electron Transport Layer. Adv. Mater. Interfaces.

[13] Yoon S.-Y., Lee Y.-J., Yang H., Jo D.-Y., Kim H.-M., Kim Y., Park S.M., Park S., Yang H. (2022). Performance Enhancement of InP Quantum Dot Light-Emitting Diodes via a Surface-Functionalized ZnMgO Electron Transport Layer. ACS Energy Lett..

[14] Chen M., Li Q., Bian Y., Wang S., Hu B., Tang A., Chen F., Lv Y., Shen H. (2025). High-Efficiency and Stable Green InP-QLED Enabled by Lowering Electron Injection Barrier. Adv. Opt. Mater..

[15] Kim D.C., Seung H., Yoo J., Kim J., Song H.H., Kim J.S., Kim Y., Lee K., Choi C., Jung D. (2024). Intrinsically stretchable quantum dot light-emitting diodes. Nat. Electron..

[16] Cao F., Wu Q., Zhang S., Yu W., Kong L., Yang Y., Zhang J., Wang S., Yang X. (2025). Surface- and Spatial-Regulated Cd-Free Quantum Dots for Efficient, Mechanically Stable, and Full-Color Flexible Light-Emitting Diodes. Adv. Mater..

[17] Bi Y., Sun J., Cao S., Li Q., Zheng J., Yuan X., Wang Y., Zou B., Zhao J. (2025). Highly efficient and eco-friendly green quantum dot light-emitting diodes through interfacial potential grading. Nat. Commun..

[18] Won Y.-H., Cho O., Kim T., Chung D.-Y., Kim T., Chung H., Jang H., Lee J., Kim D., Jang E. (2019). Highly efficient and stable InP/ZnSe/ZnS quantum dot light-emitting diodes. Nature.

[19] Chaudhary A., Shukla R.K., Malik P., Mehra R., Raina K.K. (2019). ZnO/FLC nanocomposites with low driving voltage and non-volatile memory for information storage applications. Curr. Appl Phys..

[20] Awasthi S., Pramanik S., Singh K.K., Mohan A., Pal B.N. (2024). Highly Flexible Non-Volatile Resistive Memory Devices Based on ZnO Nanoparticle/Graphene Heterostructures Embedded in Poly(methyl methacrylate). ACS Appl. Nano Mater..

[21] Chen G., Song C., Chen C., Gao S., Zeng F., Pan F. (2012). Resistive Switching and Magnetic Modulation in Cobalt-Doped ZnO. Adv. Mater..

[22] Patil V.L., Patil A.A., Patil S.V., Khairnar N.A., Tarwal N.L., Vanalakar S.A., Bulakhe R.N., In I., Patil P.S., Dongale T.D. (2020). Bipolar resistive switching, synaptic plasticity and non-volatile memory effects in the solution-processed zinc oxide thin film. Mater. Sci. Semicond. Process..

[23] Park Y., Han U.-B., Kim M.-K., Lee J.-S. (2018). Solution-Processed Flexible Threshold Switch Devices. Adv. Electron. Mater..

[24] Carlos E., Branquinho R., Martins R., Kiazadeh A., Fortunato E. (2021). Recent Progress in Solution-Based Metal Oxide Resistive Switching Devices. Adv. Mater..

[25] Belmoubarik M., Al-Mahdawi M., Machado G., Nozaki T., Coelho C., Sahashi M., Peng W.K. (2024). Resistive switching and Schottky barrier modulation at CoPt/ferroelectric-like MgZnO interface for non-volatile memories. J. Mater. Sci. Mater. Electron..

[26] Zhang H., Alanthattil A., Webster R.F., Zhang D., Ghasemian M.B., Venkataramana R.B., Seidel J., Sharma P. (2023). Robust Switchable Polarization and Coupled Electronic Characteristics of Magnesium-Doped Zinc Oxide. ACS Nano.

[27] Kumar M., Dhar J.C. (2025). Low interface state density and large capacitive memory window using RF sputtered NiO nanoparticles decorated MgZnO thin film. Sci. Rep..

[28] She Y., Peng Y., Tang B., Hu W., Qiu J., Tang X., Bao D. (2018). Bipolar resistive switching effects with self-compliance and multilevel storage characteristics in Ag/MgZnO/Si structures. Ceram. Int..

[29] Heo S.B., Shin J.S., Kim T.Y., Park S., Jung W.H., Kim H., Hong J.-A., Kim B.-S., Park Y., Chin B.D. (2021). Highly efficient and low turn-on voltage quantum-dot light-emitting diodes using a ZnMgO/ZnO double electron transport layer. Curr. Appl Phys..

[30] Lee K., Lee J., Bae Y., Roh H., Jung W.H., Lim J., Kim J., Roh J. (2024). Interfacial Modification of ZnO/ZnMgO Bilayer for Efficient and Stable InP Quantum Dot Light-Emitting Diodes via Ultraviolet Ozone Treatment. ACS Appl. Mater. Interfaces.

[31] Sun Y., Jiang Y., Peng H., Wei J., Zhang S., Chen S. (2017). Efficient quantum dot light-emitting diodes with a Zn_0.85_Mg_0.15_O interfacial modification layer. Nanoscale.

[32] Chen L., Qin Z., Chen S. (2022). Ultrahigh Resolution Pixelated Top-Emitting Quantum-Dot Light-Emitting Diodes Enabled by Color-Converting Cavities. Small Methods.

[33] Azeem W., Su S., Ho L.P., Younas M., Azad F., Rashid R. (2019). Point contact bipolar resistive switching observed in transparent ZnMgO/ZnO:Ga heterostructure. J. Mater. Sci. Mater. Electron..

[34] Chen X., Hu W., Wu S., Bao D. (2014). Complementary switching on TiN/MgZnO/ZnO/Pt bipolar memory devices for nanocrossbar arrays. J. Alloys Compd..

[35] Chen X., Hu W., Wu S., Bao D. (2014). Stabilizing resistive switching performances of TiN/MgZnO/ZnO/Pt heterostructure memory devices by programming the proper compliance current. Appl. Phys. Lett..

[36] Yoon J.-G. (2020). A New Approach to the Fabrication of Memristive Neuromorphic Devices: Compositionally Graded Films. Materials.

[37] Ding S., Wu Z., Qu X., Tang H., Wang K., Xu B., Sun X.W. (2020). Impact of the resistive switching effects in ZnMgO electron transport layer on the aging characteristics of quantum dot light-emitting diodes. Appl. Phys. Lett..

[38] Xia Y., He W., Chen L., Meng X., Liu Z. (2007). Field-induced resistive switching based on space-charge-limited current. Appl. Phys. Lett..

[39] Deshmukh A.P., Patil K., Ogale S., Bhave T. (2023). Resistive Switching in CsPbBr_3_ (0D)/MoS_2_ (2D) Heterojunction System: Trap-Controlled Space Charge Limited Transport Mechanism. ACS Appl. Electron. Mater..

[40] Harada T., Ohkubo I., Tsubouchi K., Kumigashira H., Ohnishi T., Lippmaa M., Matsumoto Y., Koinuma H., Oshima M. (2008). Trap-controlled space-charge-limited current mechanism in resistance switching at Al/Pr0.7Ca0.3MnO3 interface. Appl. Phys. Lett..

[41] Kim D., Bang H., Baac H.W., Lee J., Truong P.L., Jeong B.H., Appadurai T., Park K.K., Heo D., Nam V.B. (2023). Room-Temperature-Processable Highly Reliable Resistive Switching Memory with Reconfigurability for Neuromorphic Computing and Ultrasonic Tissue Classification. Adv. Funct. Mater..

[42] Sun Y., Han C., Li R., Xiang C., Zhang T., Qian L. (2024). Fully solution-processed red tandem quantum dot light-emitting diodes with an EQE exceeding 35%. J. Mater. Chem. C.

[43] Sun Y., Seo J.H., Takacs C.J., Seifter J., Heeger A.J. (2011). Inverted Polymer Solar Cells Integrated with a Low-Temperature-Annealed Sol-Gel-Derived ZnO Film as an Electron Transport Layer. Adv. Mater..

[44] Luo Y., Peng X., Yu S. MLP+NeuroSimV3.0: Improving On-chip Learning Performance with Device to Algorithm Optimizations. Proceedings of the International Conference on Neuromorphic Systems, Association for Computing Machinery.

[45] Buckeridge J., Catlow C.R.A., Farrow M.R., Logsdail A.J., Scanlon D.O., Keal T.W., Sherwood P., Woodley S.M., Sokol A.A., Walsh A. (2018). Deep vs shallow nature of oxygen vacancies and consequent n-type carrier concentrations in transparent conducting oxides. Phys. Rev. Mater..

[46] Lai Y., Xin P., Cheng S., Yu J., Zheng Q. (2015). Plasma enhanced multistate storage capability of single ZnO nanowire based memory. Appl. Phys. Lett..

[47] Park J.H., Alshammari F.H., Wang Z., Alshareef H.N. (2016). Interface Engineering for Precise Threshold Voltage Control in Multilayer-Channel Thin Film Transistors. Adv. Mater. Interfaces.

[48] Kim J., Jung H., Song J., Kim K., Lee C. (2017). Analysis of Interfacial Layer-Induced Open-Circuit Voltage Burn-In Loss in Polymer Solar Cells on the Basis of Electroluminescence and Impedance Spectroscopy. ACS Appl. Mater. Interfaces.

[49] Eun Y.-B., Jang G.-P., Yang J.-H., Kim S.-Y., Chae Y.-B., Ha M.-Y., Moon D.-G., Kim C.-K. (2023). Performance Improvement of Quantum Dot Light-Emitting Diodes Using a ZnMgO Electron Transport Layer with a Core/Shell Structure. Materials.

[50] Youn W., Lee J.W., Yu H., Kim D.Y. (2020). Effect of Refractive Index Contrast on Out-Coupling Efficiency of Corrugated OLEDs using Low-Refractive-Index LiF Interlayer. ACS Appl. Electron. Mater..

[51] Kwon S., Kim M.-J., Chung K.-B. (2021). Multi-level characteristics of TiOx transparent non-volatile resistive switching device by embedding SiO2 nanoparticles. Sci. Rep..

[52] Huang C.-Y., Huang C.-Y., Tsai T.-L., Lin C.-A., Tseng T.-Y. (2014). Switching mechanism of double forming process phenomenon in ZrOx/HfOy bilayer resistive switching memory structure with large endurance. Appl. Phys. Lett..

[53] Tsai T.-L., Chang H.-Y., Lou J.J.-C., Tseng T.-Y. (2016). A high performance transparent resistive switching memory made from ZrO2/AlON bilayer structure. Appl. Phys. Lett..

[54] Mahata C., Kang M., Kim S. (2020). Multi-Level Analog Resistive Switching Characteristics in Tri-Layer HfO_2_/Al_2_O_3_/HfO_2_ Based Memristor on ITO Electrode. Nanomaterials.

[55] Popov A.I., Kotomin E.A., Maier J. (2010). Basic properties of the F-type centers in halides, oxides and perovskites. Nucl. Instrum. Methods Phys. Res. Sect. B.

[56] McCluskey M.D., Jokela S.J. (2009). Defects in ZnO. J. Appl. Phys..

[57] Ghorbani A., Chen J., Chun P., Lyu Q., Cotella G., Aziz H. (2024). Changes in Hole and Electron Injection under Electrical Stress and the Rapid Electroluminescence Loss in Blue Quantum-Dot Light-Emitting Devices. Small.

[58] Lee J., Yang K., Kwon J.Y., Kim J.E., Han D.I., Lee D.H., Yoon J.H., Park M.H. (2023). Role of oxygen vacancies in ferroelectric or resistive switching hafnium oxide. Nano Converg..

[59] Su Q., Sun Y., Zhang H., Chen S. (2018). Origin of Positive Aging in Quantum-Dot Light-Emitting Diodes. Adv. Sci..

[60] Han S.W., Park C.J., Shin M.W. (2022). The role of Al atoms in resistive switching for Al/ZnO/Pt Resistive Random Access Memory (RRAM) device. Surf. Interfaces.

[61] Zhang K., Jia X., Cao K., Wang J., Zhang Y., Lin K., Chen L., Feng X., Zheng Z., Zhang Z. (2022). High On/Off Ratio Spintronic Multi-Level Memory Unit for Deep Neural Network. Adv. Sci..

[62] Choi S., Shin J., Park G., Eo J.S., Jang J., Yang J.J., Wang G. (2024). 3D-integrated multilayered physical reservoir array for learning and forecasting time-series information. Nat. Commun..

[63] Castro W., Oblitas J., Santa-Cruz R., Avila-George H. (2017). Multilayer perceptron architecture optimization using parallel computing techniques. PLoS ONE.

