# Supplementary material for: Resistive Switching Behavior of Sol–Gel-Processed ZnMgO/ZnO Bilayer in Optoelectronic Devices
Source: Nanomaterials (Basel). 2025 Sep 3;15(17):1353. doi: 10.3390/nano15171353 (PMC12430625; doi:10.3390/nano15171353)
Supplement: Supplementary file 1 [file nanomaterials-15-01353-s001.zip › nanomaterials-3794535-supplementary.pdf]

Supporting Information

Resistive Switching Behavior of Sol–Gel-Processed ZnMgO/ZnO Bilayer in Optoelectronic Devices

Hee Sung Shin [1], *Dong Hyun Kim* [2], *Donggu Lee* [2] *and Jaehoon Kim* [1,*]

[1]Department of Electronic Engineering, Gachon University, Seongnam-si, Gyeonggi-do, 13120, Republic of Korea

[2]Department of Semiconductor Engineering, Gyeongsang National University, Jinju-si, Gyeongsang-nam-do, 37160, Republic of Korea

[*]Correspondence: jaehoonkim@gachon.ac.kr

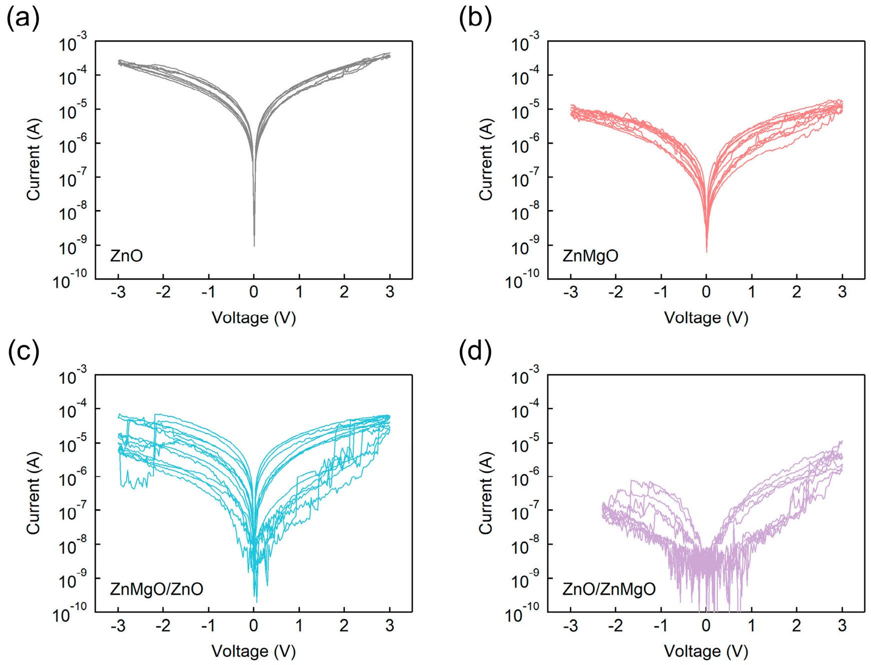

Figure S1. Device-to-device reproducibility of resistive switching characteristics: overlaid I–V curves measured from multiple devices based on (a) ZnO (b) ZnMgO (c) ZnMgO/ZnO and (d) ZnO/ZnMgO structures.

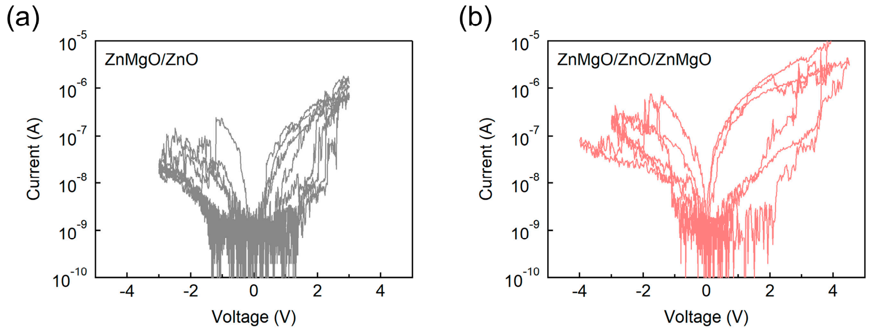

Figure S2. Device-to-device reproducibility of resistive switching characteristics: overlaid I–V curves measured from multiple devices based on (a) ZnMgO/ZnO bilayer and (b) ZnMgO/ZnO/ZnMgO trilayer structures.

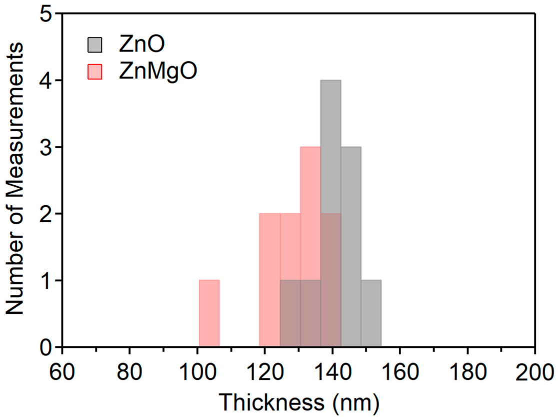

Figure S3. Thickness distribution of ZnO and ZnMgO films measured by alpha-step profilometry at multiple evenly distributed points across the substrate.
